# Positive Feedback Regulation of Circular RNA Hsa_circ_0000566 and HIF-1α promotes Osteosarcoma Progression and Glycolysis Metabolism

**DOI:** 10.14336/AD.2022.0826

**Published:** 2023-04-01

**Authors:** Shuying Shen, Yining Xu, Zhe Gong, Teng Yao, Di Qiao, Yizhen Huang, Zhenlei Zhang, Jun Gao, Haonan Ni, Zhanping Jin, Yingchun Zhu, Hongfei Wu, Qingxin Wang, Xiangqian Fang, Kangmao Huang, Jianjun Ma

**Affiliations:** ^1^Department of Orthopaedic Surgery, Sir Run Run Shaw Hospital, Zhejiang University School of Medicine, Zhejiang, China.; ^2^Key Laboratory of Musculoskeletal System Degeneration and Regeneration Translational Research of Zhejiang Province, Zhejiang, China.; ^3^Department of Orthopedic Surgery, 920th Hospital of Joint Logistics Support Force of the Chinese People’s Liberation Army, Kunming, China.; ^4^Department of Orthopedics, Ningbo First Hospital, Ningbo, Zhejiang, China.; ^5^Departments of Orthopedics, Marine Police Hospital, Zhejiang, China.

**Keywords:** Circular RNA, hypoxia, Warburg effect, hypoxia-inducible factor-1α, osteosarcoma

## Abstract

Hypoxia is an indispensable factor for cancer progression and is closely associated with the Warburg effect. Circular RNAs (CircRNA) have garnered considerable attention in molecular malignancy therapy as they are potentially important modulators. However, the roles of circRNAs and hypoxia in osteosarcoma (OS) progression have not yet been elucidated. This study reveals the hypoxia-sensitive circRNA, Hsa_circ_0000566, that plays a crucial role in OS progression and energy metabolism under hypoxic stress. Hsa_circ_0000566 is regulated by hypoxia-inducible factor-1α (HIF-1α) and directly binds to it as well as to the Von Hippel-Lindau (VHL) E3 ubiquitin ligase protein. Consequentially, binding between VHL and HIF-1α is impeded. Furthermore, Hsa_circ_0000566 contributes to OS progression by binding to HIF-1α (while competing with VHL) and by confers protection against HIF-1α against VHL-mediated ubiquitin degradation. These findings demonstrate the existence of a positive feedback loop formed by HIF-1α and Hsa_circ_0000566 and the key role they play in OS glycolysis. Taken together, these data indicate the significance of Hsa_circ_0000566 in the Warburg effect and suggest that Hsa_circ_0000566 could be a potential therapeutic target to combat OS progression.

Osteosarcoma (OS) is a highly malignant bone tumor and a commonly reported type of solid cancer in children and adolescents [1]. OS originates from mesenchymal cells and often metastasizes during the early stages [2]. Metastasis occurs in 15%-20% of all patients with OS [3, 4]. The most common forms of OS metastasis involve the lungs (85%-90%), followed by the bones (8%-10%), and occasionally the lymph nodes [5-7]. Currently, the main treatment for OS consists of a combination of chemotherapy, surgery, auxiliary radiation therapy, and biological therapy [8, 9]. However, the incidence of pulmonary metastasis is extremely high in OS [10-12]. Approximately 80% of all patients who undergo radical surgery present with lung metastases [13]. Hence, the five-year survival rate is low [14, 15]. Moreover, the rapid growth of solid tumors such as OS is often accompanied by the creation of a hypoxic microenvironment creation that promotes metastasis [16, 17].

Hypoxia-inducible factor 1-alpha (HIF-1α) encodes the α subunit of the hypoxia-inducible factor-1 (HIF-1) transcription factor (TF) [18]. HIF-1 is a heterodimer composed of an α subunit and a β subunit [19]. HIF-1 activates the transcription of several genes that are involved in the regulation of energy metabolism, angiogenesis, apoptosis, and the production of proteins that enhance oxygen delivery and metabolic adaptation to hypoxia [20]. HIF-1α is the main regulator of cellular homeostasis and the organismal responses to hypoxia [21]. Therefore, HIF-1 plays vital roles in embryonic and tumor angiogenesis, and in the pathophysiology of ischemic diseases. Under normoxic conditions, 402 and/or 564 proline residues in HIF-1α are hydroxylated by the proline hydroxylase domain (PHD) protein [22]. Under constant oxygen supply, HIF-1α with hydroxylated prolines binds to the Von Hippel-Lindau (VHL) E3 ubiquitin ligase protein which, in turn, triggers the ubiquitin-proteasome proteolysis pathway and causes its rapid degradation [23]. Under hypoxic conditions of hypoxia, HIF-1α accumulates in cells and binds to HIF-1β to form HIF-1 [24]. Thereafter, HRE-HIF-1 binding occurs and this complex participates in multiple signal transduction pathways and the cellular hypoxia response generation.

HIF-1α activates several enzymes associated with glucose metabolism and glycolysis [25]. Additionally, HIF-1α induces the glucose vector GLU and GLU-1-3 associated with glycolytic enzymes such as HK2 and aldolase A/c [26]. When the oxygen level is insufficient, HIF-1 upregulates the expression of these enzymes and facilitates adaptation to cellular hypoxia via the Warburg effect [27]. The Warburg effect (also known as aerobic glycolysis) is a typical characteristic of tumor cells, which exhibits the feature of giving priority to high glycolysis for energy supply, even in the existence of adequate oxygen. Additionally, HIF-1α participates in normal bone development and bone repair [28]. HIF-1α expression level is normally stable but is upregulated in OS osteoblasts (OB) [29]. Hence, HIF-1α presents a promising molecular target for the prevention and treatment of OS.

Non-coding RNAs (ncRNAs) are strongly associated with the occurrence and progression of various human diseases [30]. In tumor cells, LncRNAs modify glycolysis and regulate HIF-1α expression [31], whereas in cervical cancer cells, lncRNA, *IDH1-AS1*, establishes an association of MYC proto-oncogene, BHLH transcription factor (c-MYC), and HIF-1 with mitochondrial respiration and glycolysis regulation via the actions of isocitrate dehydrogenase (NADP (+) 1 and cytoplasmic (IDH1) [32]. *IDH1-AS1* expression restoration is a potential metabolic approach for cervical cancer treatment. LnRNAs such as *LncRNA-TUG1* and *LncRNA-MIF* participate in glycolysis and regulate glycolysis metabolism via competitive endogenous RNA (ceRNA) networks [33-35].

Circular RNA (circRNA) is a recently discovered ncRNA that is a new research hot spot [36]. Unlike linear RNA, circRNA has a closed-loop structure and is unaffected by ambient RNA enzyme levels [37, 38]. Hence, circRNA expression is stable and it is not easily degraded. CircRNAs are produced by selective splicing and occurs in the cytoplasm of a wide range of eukaryotic cells [39]. CircRNA biosynthesis is highly organized and precisely timed, and it exhibits disease specificity. Therefore, circRNAs have considerable potential as novel diagnostic markers.

However, the mechanisms by which circRNAs regulate the Warburg effect are unclear. It is unknown whether circRNAs assist Warburg effect regulation through hypoxia/HIF-1α. In this study, we demonstrate that the novel HIF-1α-inducible circRNA, circRNA-circ_0000566, regulates hypoxia-induced glycolysis. Furthermore, we elucidate the hypoxia/HIF-1α mechanism of the Warburg effect and indicate that Hsa_circ_0000566 may regulate this process.

## MATERIALS AND METHODS

### Cell culture

Human hFOB1.19 osteoblasts, HEK-293, and various osteosarcoma cell lines, including 143B, HOS, MG-63, and U2OS, were purchased from the American Type Culture Collection (ATCC; Manassas, VA, USA). All cells were cultivated in the Dulbecco’s modified Eagle medium (DMEM) added with 10% (v/v) fetal bovine serum (FBS; Gibco, Grand Island, NY, USA). The cells were cultured at 37 °C in a humidifying incubator under a 5% CO_2_ atmosphere. For the hypoxic or normoxic treatment, cells were cultured under hypoxic or normoxic stress for 24 hours. Treated cells were harvested for further experiments.

### Ethical approval

All animal experimentations were carried out in compliance with the principles outlined by the Guide for the Care and Use of Laboratory Animals published by the National Institutes of Health and performed under the guidelines of the Ethics Committee of Sir Run Run Shaw Hospital.

### Clinical samples

Twelve pairs of clinical osteosarcomas and chondroma samples were obtained from patients who had undergone radical resection at Sir Run Run Shaw Hospital between April 2013 and September 2017. All processes were confirmed by the Institutional Review Board of Sir Run Run Shaw Hospital and were conducted in accordance with the Declaration of Helsinki. All samples were histologically authenticated by pathologists based on the World Health Organization criteria. Informed written consent was provided by all patients prior to study initiation. The basic information of patients was exhibited in Supplementary Table 2.

### RNA isolation and quantitative real-time polymerase chain reaction (qRT-PCR)

Total RNA extraction was performed with the TRIzol reagent (Invitrogen, Carlsbad, CA, USA) from certain clinical tumor samples or osteosarcoma cells according to the manufacturer’s instructions. The extraction of cytoplasmic RNA and nuclear RNA from OS cells were performed using the Nuclear/Cytosol Fractionation Kit (Invitrogen, Carlsbad, CA, USA) according to the manufacturer’s instructions. The RNA samples were stored at -80 ?. The PrimeScript RT reagent kit (TaKaRa Bio Inc., Kusatsu, Shiga, Japan) and SYBR Premix Ex Taq II (TaKaRa Bio Inc., Kusatsu, Shiga, Japan) were used to analyze the mRNA and circRNA levels. The β-actin expression level was considered the reference standard against which the mRNA and circRNA levels were compared. The expression of circRNA and mRNA levels were calculated by using the 2^-∆∆Ct^ method. Nuclear circRNA expression was normalized against U6 expression. Primer sequences are shown in Supplementary Table 1.

### Western blotting

Treated OS cells were subjected to lysis with the radioimmunoprecipitation assay (RIPA) lysis buffer (Beyotime, Shanghai, China). Protein concentrations were determined with a bicinchoninic acid (BCA) kit (Beyotime, Shanghai, China). Identical protein quantities were separated via sodium dodecyl sulfate-polyacrylamide gel electrophoresis (SDS-PAGE). The electrophoresed protein samples were transferred onto a polyvinylidene fluoride (PVDF) membrane which was then treated with 4% skim milk in Tris-buffered saline with Tween (TBST). The membrane was then incubated with anti-LDHA antibody ab52488 (1:1000; Abcam, Cambridge, UK), anti-HIF-1α antibody ab179483 (1:1000; Abcam, Cambridge, UK), anti-VHL antibody ab270968 (1:1000; Abcam, Cambridge, UK), anti-PDK1 antibody ab202468 (1:1000; Proteintech Group Inc., Rosemont IL, USA), anti-PDK4 antibody ab110336 (1:1000; Proteintech Group Inc., Rosemont IL, USA), anti-GLUT1 antibody ab115730 (1:1000; Abcam, Cambridge, UK), anti-GLUT4 antibody ab188317 (1:1000; Abcam, Cambridge, UK), anti-ubiquitin antibody ab134953 (1:1000; Abcam, Cambridge, UK), and anti-β-actin antibody AF2811 (1:1000; Beyotime, Shanghai, China) at 4 ? overnight. The next day, the membrane was subjected to treatment with secondary antibodies FD0114, FD0115 (Fudebio, Hangzhou, China). The membrane was then washed with TBST, and the signal intensity was measured by using FDbio-Femto enhanced chemiluminescence (ECL; Fudebio, Hangzhou, China).

### RNA pulldown

After the OS cells were transfected or subjected to treatment, 2.5 μg of each OS cell extract was mixed with 2 μg control probe or circular RNA probe for 2 h. The mixtures were incubated with 50 μL treptavidin agarose beads (Invitrogen, Carlsbad, CA, USA) for 1.5 h. The beads were then washed thrice, and the protein expression level was examined by conducting western blotting.

### RNA immunoprecipitation (RIP) experiment

The RIP assay was conducted using the Magna RIP RNA-binding protein immunoprecipitation kit (EMD Millipore, Billerica, MA, USA) in accordance with the manufacturer’s instructions. Pretreated OS cells cultured at 85% confluence were harvested utilizing RIP lysis buffer added with RNase and protease inhibitors. Each cell extract (150 μL) was subjected to treatment with the RIP buffer containing the magnetic beads bound to the aforementioned antibodies. Mouse anti-IgG antibody was used for normalization.

### Subcutaneous and orthotopic xenograft tumor models

Nude mice (male; age: 4-5 weeks) were injected with 1 × 10^7^ stably transfected 143B cells either subcutaneously or in the tibial cavity. Tumor volumes were calculated according to Equation (1): Volume (mm^3^) = ab^2^/^2^ (1).

Four weeks after treatment, the mice were sacrificed, and their tumors were harvested and weighed. Tumor samples were fixed in 4% (v/v) paraformaldehyde (PFA).

### Tail vein metastasis and bioluminescence photography

Luminescence- and green fluorescent protein (GFP)-labeled OS cells were stably transfected with Luc-vector, Luc-Hsa_circ_0000566-silencing, or Luc-Hsa_circ_ 0000566-silencing and HIF-1α-overexpressing vectors. Nude mice (age: 4-5 weeks) were subjected to injection in the tail vain with equal amounts of three different stable OS cells. Thirty days after injection, lung metastasis progress was evaluated by conducting in vivo bioluminescence photography.

### Cell vitality detection, Transwell™ migration assay, and cell apoptosis rate analysis

Cell vitality was detected employing the Cell Counting Kit (CCK)-8 (Dojindo Laboratories Inc., Kumamoto, Japan) according to the manufacturer’s protocols. Transwell™ migration assays were performed in Transwell™ chambers (BD Biosciences, NJ, USA). Cell apoptosis rates were measured by conducting flow cytometry (BD FACSCanto™ II; BD Biosciences, Franklin Lakes, NJ, USA). The FlowJo software (FlowJo LLC, Ashland, OR, USA) was employed to analyze the result.

### EdU assay

The proliferation capacities of OS cells were measured using the EdU Cell Proliferation Detection Kit (Beyotime, Shanghai, China). 48 hours after transfection, OS cells were co-incubated with 500μL Click Reaction Buffer and were incubated for approximately 30 min. To measure the percentage of cell proliferation, Hoechst 33342 were employed for nuclear staining. Images were captured utilizing a fluorescence microscope. Representative images were selected at least three random fields of view.

### Soft agar colony formation experiment

Transfected OS cells were cultured in semisolid agar medium in 6-well plates at a density of 15000 cells/well. The cells were cultured for 1, 7, 14 days and representative pictures of were taken under a microscope ((Zeiss, Primovert).

### Ubiquitination immunoprecipitation experiments

Ubiquitination immunoprecipitation experiment was carried out utilizing an Immunoprecipitation Kit (Beyotime, Shanghai, China). Proteins in transfected OS cells were extracted with RIPA lysis buffer (Beyotime, Shanghai, China). Magnetic beads were washed and were added with 5μL of HIF-1α or IgG antibody. After incubation, a beads-Ab complex was obtained and was incubated with antigen samples to form a beads-Ab-Ag complex. The immune complex eluted from magnetic beads was used for western blotting and the ubiquitination of different samples were detected.

### Colony formation assay

Identical amounts of transfected 143B and HOS cells were seeded in 12-well plates at a density of 700 cells/well. Fifteen days after seeding, the colonies were subjected to fixation with 4% (v/v) PFA for 15 min followed by staining with 0.5% (w/v) crystal violet for 15 min. The colonies were enumerated and photographed under a microscope (Zeiss, Primovert).

### RNA in situ hybridization

Alexa Fluor 555-labeled Hsa_circ_0000566 probes were purchased from RiboBio (Guangzhou, China). The probe sequences are validated upon requirement. The probe signals were detected with a fluorescent *in situ* hybridization (FISH) kit (RiboBio, Guangzhou, China). Images were captured using the Nikon A1Si confocal laser scanning microscope (CLSM; Nikon Instruments Inc., Tokyo, Japan).

### Glucose uptake, lactate production, and ATP concentration

The experimental procedures used in the current research to determine glucose uptake, lactate production, and ATP concentration have been reported in previous studies. Glucose was detected using a glucose assay kit (BioVision, Inc., Milpitas, CA, USA). Lactate levels were determined using a lactate colorimetric experiment kit (BioVision, Inc., Milpitas, CA, USA). ATP concentrations were examined using an ATP determination kit (Thermo Fisher Scientific, Waltham, MA, USA).

### Chromatin immunoprecipitation (ChIP) assay

ChIP assays were conducted with an EZ-ChIP kit (EMD Millipore, Billerica, MA, USA) according to the manufacturer’s instructions.

### Immunohistochemistry

In brief,after dewaxing, rehydration and incubation, slides were blocked in 10% normal goat serum for approximately 20 min. Then, slides were incubated with LDHA, PDK1, PDK4, GLUT1 and GLUT 4 at 4 °C overnight. On the following day, Samples were washed with PBS and incubated with the second antibody for 50 min at room temperature. Immunoreactivity was detected using the (DAB Horseradish Peroxidase Color Development Kit (Beyotime, Shanghai, China). Iisotype antibody controls, secondary antibody only controls, tissue minus/plus controls were employed to validate antibody specificity and distinguish genuine target staining from background.

**Figure 1.**
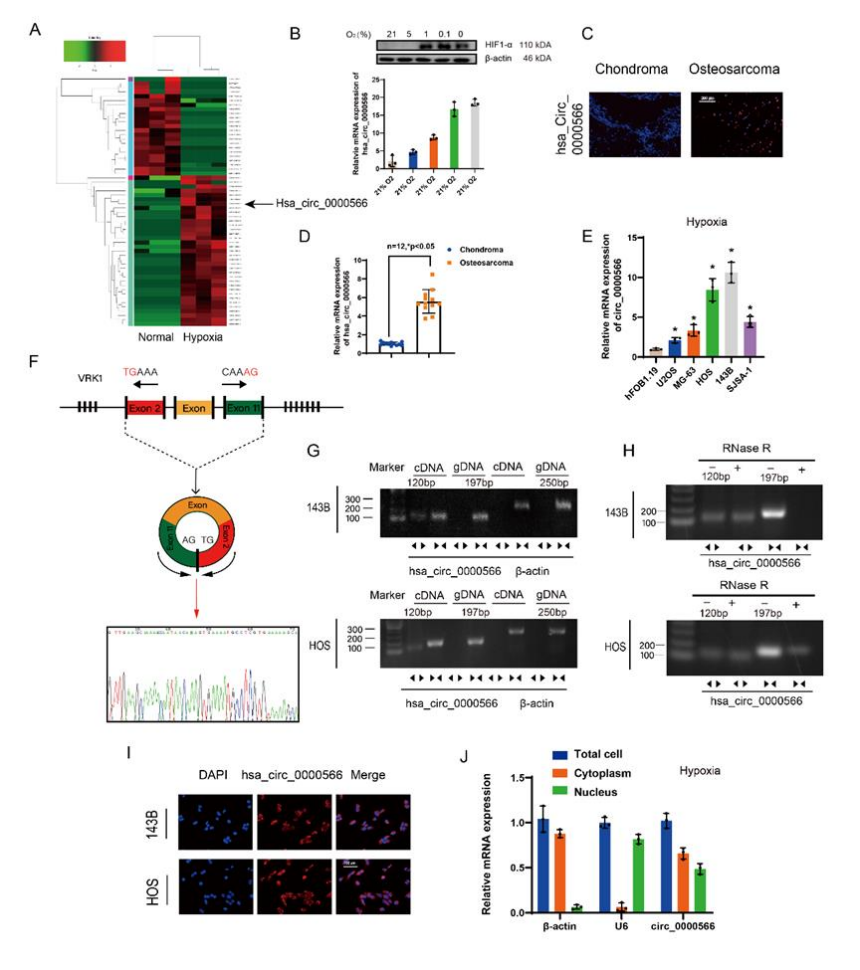
Hypoxia-associated circRNA profiling and expression characteristics of Hsa_circ_0000566 in osteosarcoma (OS). (A) CircRNA microarray analysis reveals 35 upregulated and 23 downregulated circRNAs in OS cells under normoxic and hypoxic conditions. The black arrow represents Hsa_circ_0000566. (B) OS cells incubated under various oxygen concentrations. Total RNA extraction was performed for qRT-PCR assay. Western blotting was performed to determine the protein level of HIF-1α. Results are reported as mean ± standard deviation (SD), *p < 0.05, n = 3. Scale bars, 200 μm. (C) Hsa_circ_0000566 expression is much higher in primary OS tissue than in chondroma tissue. Results are representative images according to three different experiments. (D) Quantitative real-time polymerase chain reaction (qRT-PCR) results comparing Hsa_circ_0000566 mRNA expression in 12 OS and chondroma samples. Results are reported as mean ± SD, *p < 0.05, n = 12. (E) Hsa_circ_0000566 expression levels in hFOB1.19 and various OS cell lines. Results are reported as mean ± SD, *p < 0.05, n = 3. (F) Schematic diagram showing Hsa_circ_0000566 back-spliced by exons 2-11 of the VRK1 gene and the corresponding Sanger sequencing. (G) RT-PCR results validating the presence of Hsa_circ_0000566 in 143B and HOS cells. Various primers amplified the Hsa_circ_0000566 region in cDNA but not in genomic DNA. β-actin was used as the negative control. Divergent primers are presented as the opposite direction of the arrowhead, and the convergent primers were shown as the face-to-face direction of the arrowhead. (H) RT-PCR results indicating Hsa_circ_0000566 and VRK1 mRNA expression in untreated 143B and HOS cells and in the cells subjected to treatment with RNase-R. (I) RNA fluorescence in situ hybridization (FISH) results revealing Hsa_circ_0000566 localized mainly in the cytoplasm. Hsa_circ_0000566 probes were labeled with cy3 and nuclei were stained with 4’,6-diamidino-2-phenylindole (DAPI). Scale bars, 100 μm. (J) qRT-PCR determination of the main localization of Hsa_circ_0000566 in OS cells. Results are reported as mean ± SD, *p < 0.05, n = 3.

### Statistics and data analysis

Statistical and data analyses were conducted using the SPSS software (version 22.0; IBM Corp., Armonk, NY, USA). Results are reported as the mean ± standard deviation (SD). Statistical tests were performed using non-parametric alternatives. Data from the two experimental groups were compared using the Wilcoxon signed-rank test. Experimental data from multiple groups were evaluated using the Kruskal—Wallis test. Statistical significance was defined as P < 0.05.

## RESULTS

### Hsa_circ_0000566 is overexpressed and induced by hypoxic stress in OS

We first explored potentially hypoxia-responsive circRNAs to determine the circRNAs that has a significant change in expression levels in OS cells under hypoxia. Based on the degree of differential expression, five circRNAs with high and low expression under hypoxic stress from circRNA microarray analysis were selected (Supplementary Fig. 1A and Fig. 1A). The genes for these circRNAs were knocked down in OS cells and screened by performing CCK-8, migration, and apoptosis assays (Figure S1B-D). Knockdown of Hsa_ circ_0000566 significantly reduced OS cell proliferation, migration, and viability. Moreover, the hypoxia-induced expression of Hsa_circ_0000566 was dose-dependent (Fig. 1B). Hypoxia-induced Hsa_circ_0000566 expression was detected in 143B, HOS, U2OS, MG-63, and SJSA-1 cells (Fig. 1E). These data indicate the specificity of Hsa_circ_0000566 for hypoxic induction.

Fluorescence in situ hybridization (FISH) results indicated that Hsa_circ_0000566 expression in OS was significantly higher than in chondrosarcoma (Fig. 1C). We also studied the mRNA expression of Hsa_circ_0000566 in OS and chondrosarcoma, and in 12 pairs of specimens, Hsa_circ_0000566 expression was also significantly higher in OS than in the control (Figure 1D). Additionally, quantitative real-time PCR (qRT-PCR) results showed that Hsa_circ_0000566 expression was much higher in various OS cell lines than in hFOB1.19 (Fig. 1E). Figure 1F shows that Hsa_circ_0000566 was generated from VRK1 that is located on chromosome 14. In addition, the Hsa_circ_0000566 annotation contained exons 2-11 of VRK1. RT-PCR was performed to validate the head-to-tail splicing of endogenous Hsa_circ_0000566 using divergent and convergent primers (Fig. 1G). Accordingly, only the divergent primers for Hsa_circ_0000566 amplified the PCR product, which identified the circular structure of Hsa_circ_0000566. In addition, the RNaseR tolerance test results indicated that Hsa_circ_0000566 was resistant to RNaseR treatment, whereas the PCR product of convergent primers decreased visibly after RnaseR treatment (Fig. 1H). FISH cell location and qRT-PCR revealed that Hsa_circ_0000566 was localized mainly in the cytoplasm and nucleus of OS cells (Fig. 1I-J).

### Hsa_circ_0000566 knockdown restrains OS progression in vitro

**Figure 2.**
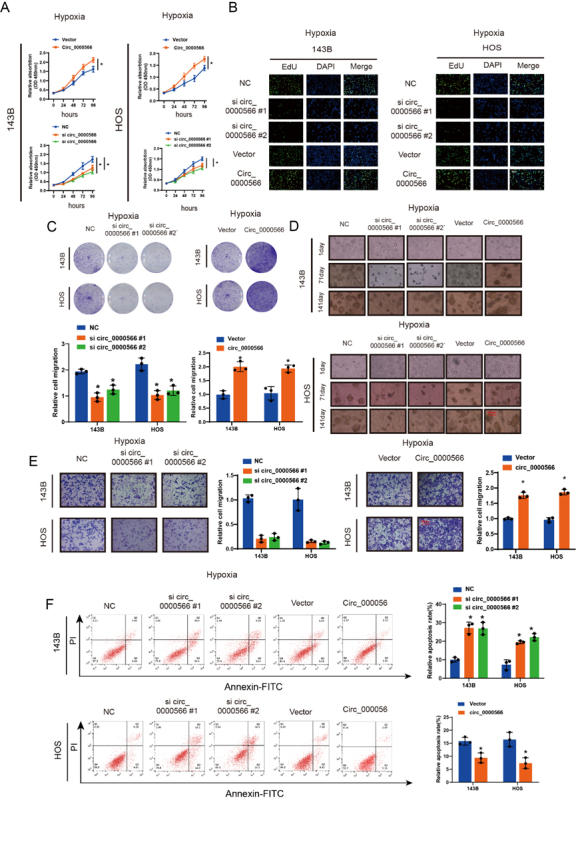
Hsa_circ_0000566 contributes to in vitro osteosarcoma (OS) cell progression under hypoxic conditions. (A) Hsa_circ_0000566 overexpression and knockdown induced and repressed OS cell proliferation under hypoxia. Results are reported as mean ± standard deviation (SD), *p < 0.05, n = 3. Circ_0000566 represents Hsa_circ_0000566 overexpression, and si circ_0000566 represents Hsa_circ_0000566 knockdown. Vector and Si NC represents the negative control of Hsa_circ_0000566 overexpression and Hsa_circ_0000566 knockdown, respectively. (B) EdU exhibits the impact of Hsa_circ_0000566 on OS cell proliferation under hypoxia. Nuclei are stained with 4’,6-diamidino-2-phenylindole (DAPI). Results are reported as mean ± SD, *p < 0.05, n = 3. Scale bars, 100 μm. (C) Colony formation experiment verifies Hsa_circ_0000566 functions in OS cells under hypoxia. Results are reported as mean ± SD, *p < 0.05, n = 3. (D) Soft agar colony formation assay indicates the effects of Hsa_circ_0000566 on 143B and HOS cell colony forming capacity under hypoxia. Results are reported as mean ± SD, *p < 0.05, n = 3. Scale bars, 100 μm. (E) OS cell migration capacity as determined by Transwell™ migration assays. Results are reported as mean ± SD, *p < 0.05, n = 3. Scale bars, 100 μm. (F) Flow cytometry verifies Hsa_circ_0000566 functions in OS cell apoptosis. Results are reported as mean ± SD, *p < 0.05, n = 3.

CCK-8 cell viability experiments were performed to verify Hsa_circ_0000566 function in OS cells (Fig. 2A). Three different Hsa_circ_0000566-targeting siRNAs were used to knockdown Hsa_circ_0000566 (Supplementary Fig. 2A). two siRNAs consistently achieved the highest Hsa_circ_0000566 knockdown efficiency. Hsa_circ_ 0000566 overexpression plasmids were also transfected into OS cells, and the overexpression efficiency was determined by qRT-PCR (Supplementary Fig. 2B). Hsa_circ_ 0000566 overexpression promoted proliferation of HOS and 143B OS cells, whereas Hsa_circ_0000566 knockdown exerted an opposite effect (Fig. 2B). Moreover, cell plate cloning experiments showed that Hsa_circ_0000566 promoted OS cell proliferation (Fig. 2C). Soft agar cloning assays were conducted to clarify the effects of Hsa_circ_0000566 on OS cell tumorigenicity (Fig. 2D). Hsa_circ_0000566 promoted the formation of soft agar OS cell clones. Hence, it enhanced the transformation capacity of OS cells. TranswellTM experiments showed that Hsa_circ_0000566 overexpression promoted OS cell migration, whereas Hsa_circ_0000566 knockdown exerted an opposite effect (Fig. 2E). Flow cytometry and double-staining apoptosis experiments revealed that Hsa_circ_0000566 inhibited apoptosis in OS cells (Fig. 2F).

### Hsa_circ_0000566 promotes glycolysis in osteosarcoma and is essential for hypoxia-enhanced glycolysis

**Figure 3.**
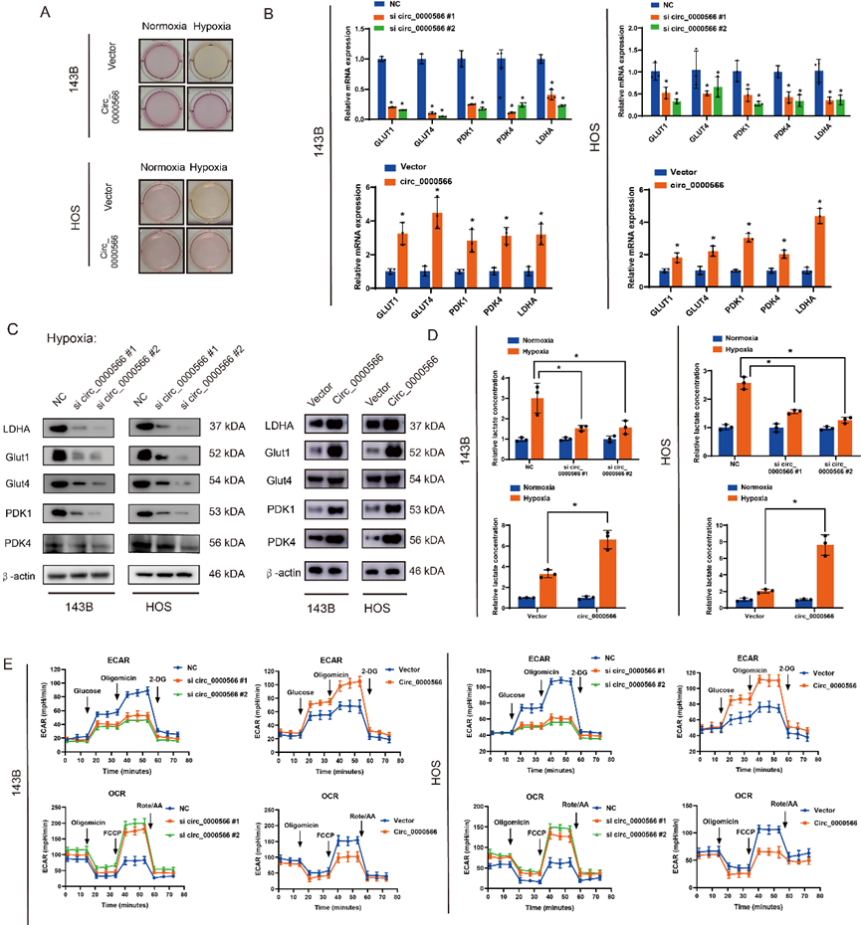
Hsa_circ_0000566 accelerates osteosarcoma (OS) glucose metabolism and regulates hypoxia-enhanced glycolysis. (A) Colors of the media indicate that Hsa_circ_0000566 silencing decreased lactate accumulation under hypoxia. (B-C) Quantitative real-time polymerase chain reaction (qRT-PCR) or western blots evaluating the expression levels of genes involved in glucose metabolism in 143B and HOS cells transfected with Hsa_circ_0000566-overexpressing, Hsa_circ_0000566 (shRNA), or vector plasmids. Results are reported as mean ± standard deviation (SD), *p < 0.05, n = 3. (D) Hsa_circ_0000566 knockdown in OS cells with decreased lactate accumulation, while Hsa_circ_0000566 overexpression has increased lactate accumulation. Results are reported as mean ± SD, *p < 0.05, n = 3. (E) Extracellular acidification rate (ECAR) indicates glycolysis rate. ECAR decreases in response to Hsa_circ_0000566 knockdown and increases in response to Hsa_circ_0000566 overexpression. Oxygen consumption rate (OCR) represented mitochondrial respiratory capacity. OCR is enhanced in response to Hsa_circ_0000566 silencing and reduced in response to Hsa_circ_0000566 overexpression in OS cells. Results are reported as mean ± SD, *p < 0.05, n = 3.

Hypoxic stress increases glucose uptake, glycolysis, and lactic acid production. These results indicated that the medium was acidified under hypoxic conditions. All hypoxia-induced effects were substantially reversed by Hsa_circ_0000566 knockdown (Fig. 3A). In addition, Hsa_circ_0000566 silencing significantly decreased glucose uptake and lactate production in OS cells under hypoxic (Supplementary Fig. 3A-B). Thus, Hsa_circ_ 0000566 may mediate hypoxia-induced increase in the glycolysis rate. qRT-PCR and protein assays showed that Hsa_circ_0000566 overexpression in turn upregulated metabolic enzyme expression, whereas Hsa_circ_ 0000566 knockdown exerted an opposite effect (Fig. 3B-C). Evaluation of the lactate production rate and SeaHorse respiratory metabolism revealed that Hsa_circ_0000566 overexpression contributed to aerobic glycolysis (Fig. 3D-E).

### Hsa_circ_0000566 establishes interactions with HIF-1α and confers protection against ubiquitination-mediating degradation

**Figure 4.**
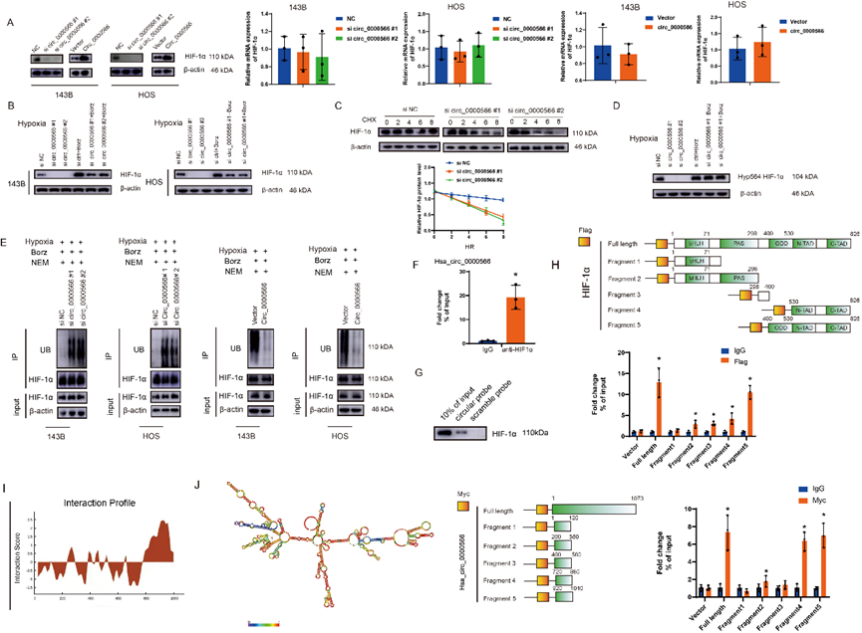
Hsa_circ_0000566 establishes interactions with HIF-1α and confers protection against ubiquitination-mediating degradation. (A) Effects of Hsa_circ_0000566 knockdown and Hsa_circ_0000566 overexpression on mRNA and protein expression in 143B and HOS cells under hypoxia. Results are reported as mean ± standard deviation (SD), *p < 0.05, n = 3. (B) Western blotting results revealing the impact of bortezomib treatment on the changes occurring at HIF-1α protein level mediated by Hsa_circ_0000566 silencing and vector transfection. (C) Western blotting assessment of the impact of CHX treatment on the variations in HIF-1α protein levels affected by Hsa_circ_0000566 silencing and vectors. Results are reported as mean ± SD, *p < 0.05, n = 3. (D) The western blot illustrates the effects of Hsa_circ_0000566 knockdown in the Hyp564 HIF-1α protein levels in the presence or absence of bortezomib treatment. (E) Immunoprecipitation assessing the HIF-1α ubiquitination levels in Hsa_circ_0000566 silencing and Hsa_circ_0000566 overexpressing osteosarcoma (OS) cells under hypoxia. Culture media were supplemented with bortezomib (250 nM) for 6 h. (F) The combination of Hsa_circ_0000566 with HIF-1α confirmed by radioimmunoprecipitation (RIP). Results are reported as mean ± SD, *p < 0.05, n = 3. (G) Pulldown assay validation of the interaction between Hsa_circ_0000566 and HIF-1α. (H) A RIP assay of HIF-1α regions interacting with Hsa_circ_0000566. Schematic diagram shows HIF-1α protein fragments. Results are reported as mean ± SD, *p < 0.05, n = 3. (I) Interaction profile between Hsa_circ_0000566 and HIF-1α obtained from catRAPID (left). (J) Schematic diagram showing Hsa_circ_0000566 RNA fragments. Combinative regions between Hsa_circ_0000566 and HIF-1α were identified by RIP assay. Results are reported as mean ± SD, *p < 0.05, n = 3.

The mechanism by which Hsa_circ_0000566 regulates glycolysis in OS involves HIF-1α expression was then investigated. qRT-PCR and western blotting was used to study the regulation of HIF-1α expression mediated by the upregulation or downregulation of Hsa_circ_0000566 (Fig. 4A). Under hypoxia, Hsa_circ_0000566 regulates HIF-1α protein levels but not HIF-1α mRNA levels. Additionally, HIF-1α reduction caused by Hsa_circ_ 0000566 knockdown was restored in the presence of the proteasome inhibitor, bortezomib (Fig. 4B). Hence, Hsa_circ_0000566 inhibits HIF-1α protein degradation under hypoxic stress. Moreover, CHX treatment reduced the protein level of HIF-1α in OS cells after Hsa_ circ_0000566 knockdown (Fig. 4C). Prolyl hydroxylase domain (PHD) enzymes catalyzed hydroxylation of the proline residue at position 564 of the oxygen-dependent degradation domain of the HIF-1α subunit. Thereafter, the hydroxylated proline residue was bound to VHL, and the latter was degraded by the ubiquitin protease. Based on previous research, hydroxylation of the HIF-1α protein at proline residue 564 is essential for VHL binding and degradation in a ubiquitin-proteasome manner. Therefore, we investigated the impact of Hsa_circ_0000566 on Hyp564 HIF-1α expression. Intriguingly, we found that Hsa_circ_0000566 knockdown under hypoxia downregulated Hyp564-HIF-1α levels; however, this effect could be restored in the presence of bortezomib (Fig. 4D). Subsequent ubiquitination immuno-precipitation experiments showed that Hsa_circ_0000566 overexpression inhibited HIF-1α ubiquitination, whereas Hsa_circ_0000566 knockdown promoted HIF-1α degradation in OS cells (Fig. 4E).

CircRNAs regulate their function by binding to their target proteins. RIP and RNA pulldown assays demonstrated that Hsa_circ_0000566 directly bound to HIF-1α (Fig. 4F-G). To determine the specific HIF-1α regions that interact with Hsa_circ_0000566 in vitro, HEK-293T cells were transfected with Hsa_circ_0000566 and vectors encoding a variety of HIF-1α protein fragments (Fig. 4H). The results showed that HIF-1α fragment 5 interacted with Hsa_circ_0000566. We believe that this may be because fragment 5 contains an ODD region of HIF-1α. To explore the interaction between Hsa_circ_0000566 and HIF-1α protein in another way, we performed Hsa_circ_0000566 segmentation according to its secondary structure, the hairpin loop. The RNA Folder website was used to identify the optimal folding of the Hsa_circ_0000566 secondary structure. We discovered that Hsa_circ_0000566 fragments 4 and 5 firmly interacted with HIF-1α (Fig. 4J), which was approximately identical to the prediction of the interaction profile obtained from the CATRAPID website (Fig. 4I).

### Hsa_circ_0000566 stabilizes HIF-1α by disrupting VHL-HIF-1α interactions in hypoxia

**Figure 5.**
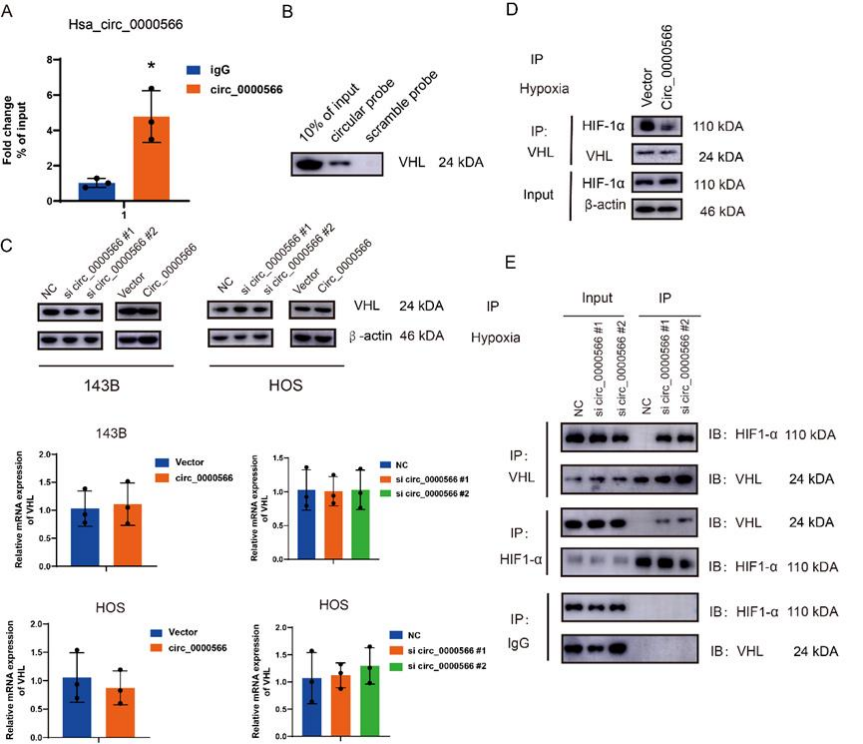
Hypoxia-induced Hsa_circ_0000566 stabilizes HIF-1α by attenuating interactions between Von Hippel—Lindau (VHL) and HIF-1α. (A) The interaction between VHL and Hsa_circ_0000566, assessed by performing a RIP assay. Results are reported as mean ± standard deviation (SD), *p < 0.05, n = 3. (B) Interaction between VHL and Hsa_circ_0000566, identified by conducting pulldown assays. (C) Effects of Hsa_circ_0000566 silencing and Hsa_circ_0000566 overexpression on VHL protein and mRNA expression, identified by performing western blotting and quantitative real-time polymerase chain reaction (qRT-PCR). (D) Immunoprecipitation experiment revealing VHL-HIF-1α interaction that is inhibited by Hsa_circ_0000566 overexpression. (E) Sequential coimmunoprecipitation assay, performed to determine whether Hsa_circ_0000566 could simultaneously bind VHL and HIF-1α.

T To determine whether Hsa_circ_0000566 could bind to VHL, RIP and RNA pulldown experiments were performed. The results indicated that Hsa_circ_0000566 could directly interact with HIF-1α and VHL (Fig. 5A-B). However, qRT-PCR and western blotting results showed that Hsa_circ_0000566 did not affect VHL expression (Fig. 5C). In contrast, immunoprecipitation experiments revealed that the overexpression of Hsa_circ_0000566 impeded the binding of VHL to HIF-1α (Fig. 5D). An additional deletion mutant assay was performed to verify this mechanism and discovered that two VHL deletion mutants could firmly interact with Hsa_circ_0000566 (amino acids 54-213 & 74-213), whereas the rest of the two VHL deletion mutants (amino acids 1-156 & 1-186) were lacking in the interaction with Hsa_circ_0000566 (Supplementary Fig. 4A-B). Based on previous studies, a single VHL deletion mutant (amino acids 54-213) possesses HIF-1α-binding capacity. Thus, Hsa_circ_ 0000566 may compete with HIF-1α for VHL binding and protect HIF-1α from VHL-dependent degradation. Additionally, we performed a sequential co-immunoprecipitation assay to determine whether Hsa_circ_0000566, VHL, and HIF-1α formed a ternary complex (Fig. 5E); Hsa_circ_0000566 could not simultaneously bind to VHL and HIF-1α.

### Hsa_circ_0000566 regulation of glycolysis under hypoxic stress depends on HIF-1α expression

**Figure 6.**
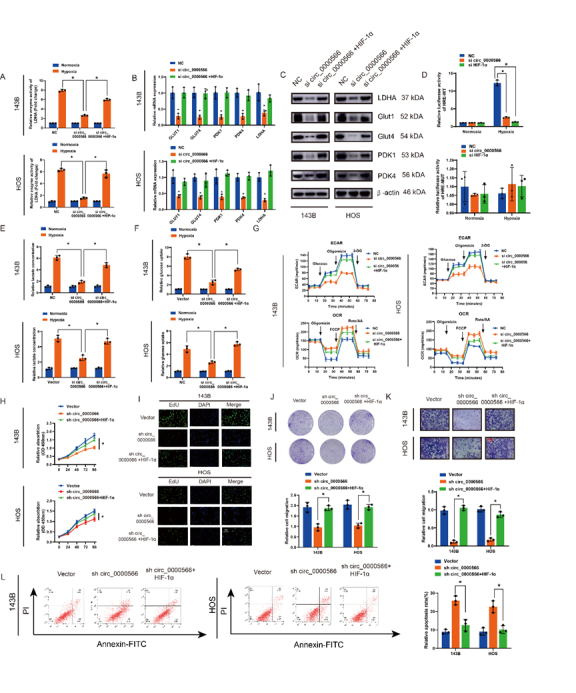
HIF-1α overexpression reverses Hsa_circ_0000566 silencing-induced attenuation of osteosarcoma (OS) cell proliferation, migration, and glucose metabolism. (A) Hsa_circ_0000566 silencing in OS cells inhibits LDHA enzyme activity, whereas HIF-1α overexpression promotes enzyme activity. (B-C) Quantitative real-time polymerase chain reaction (qRT-PCR) and western blots identifying the effects of HIF-1α overexpression in Hsa_circ_0000566 knockdown OS cells. Results are reported as mean ± standard deviation (SD), *p < 0.05, n = 3. (D) A luciferase report assay showing that Hsa_circ_0000566 knockdown markedly reversed HIF-1α transcription induced by hypoxic stress. Results are reported as mean ± SD, *p < 0.05, n = 3. (E-F) HIF-1α overexpression recovered Hsa_circ_0000566 knockdown-induced decreases in glucose uptake and lactate production. Results are reported as mean ± SD, *p < 0.05, n = 3. (G) Extracellular acidification rate (ECAR) and oxygen consumption rate (OCR) indicate that HIF-1α overexpression recovered the decline in glycolysis rate induced by Hsa_circ_0000566 knockdown under hypoxia. Results are reported as mean ± SD, *p < 0.05, n = 3. CCK-8 experiments reveal that HIF-1α and Hsa_circ_0000566 silencing affects OS cell proliferation under hypoxia. Results are reported as mean ± SD, *p < 0.05, n = 3. (I) EdU assay shows that HIF-1α and Hsa_circ_0000566 silencing influences OS cell vitality. Scale bars, 100 μm. (J) Colony formation assay indicates that colony formation ability is mediated by HIF-1α and Hsa_circ_0000566 knockdown. Results are reported as mean ± SD, *p < 0.05, n = 3. (K) Effects of HIF-1α and Hsa_circ_0000566 attenuation on tumor migration, as evidenced by Transwell™ migration assay results. Results are reported as mean ± SD, *p < 0.05, n = 3. Scale bars, 100 μm. (L) Flow cytometry evaluates the impact of HIF-1α and Hsa_circ_0000566 attenuation on OS cell apoptosis. Results are reported as mean ± SD, *p < 0.05, n = 3.

We examined the rescue effect of HIF-1α on Hsa_circ_ 0000566-induced glycolysis. Si-Hsa_circ_ 0000566#2 was used for further research owing to its high knockdown efficiency. Lactate dehydrogenase isoform A (LDHA) enzyme activity assays showed that Hsa_circ_0000566 knockdown in OS cells reduced activity, whereas HIF-1α overexpression restored this enzyme activity loss (Fig. 6A). Moreover, qRT-PCR and western blotting results revealed that HIF-1α overexpression recovered the protein and mRNA levels of glycolysis-related genes under hypoxic stress, which were decreased by Hsa_circ_0000566 knockdown (Fig. 6B-C). Furthermore, we evaluated the effect of Hsa_circ_ 0000566 on HIF-1α transcription under hypoxic conditions. The HIF-1α complex binds to the hypoxia-responsive element (HRE). HREs were designated the HRE wild-type (HRE-WT) and HRE mutation (HRE-mut). The HRE luciferase reporter system was used to determine the effects of Hsa_circ_0000566 on HIF-1α transcription (Fig. 6D). The assay showed that, compared to normoxic stress, higher lucifer activities of HRE-WT were detected under hypoxic stress, which represented an increase in HIF-1α transcription. Nevertheless, there no significant differences were detected in the transcriptional activities of HRE mutations. In addition, similar to the results of HIF-1α silencing, Hsa_circ_0000566 knockdown significantly decreased HIF-1α transcription, whereas no difference was observed in the transcriptional activity of the HRE-muts. Additionally, HIF-1α overexpression resulted in a decrease in glucose uptake and lactate production, due to Hsa_circ_0000566 knockdown (Fig. 6E-F). Furthermore, the oxygen consumption rate (OCR) and extracellular acidification rate (ECAR) assays showed that Hsa_circ_0000566 silencing increased oxygen consumption and decreased glycolysis in OS cells, and this effect was partially eliminated by HIF-1α overexpression (Fig. 6G). We selected si-circ Hsa_0000566#2 and constructed a shRNA, sh-circ _0000566. In addition, various functional assays indicated that the attenuation of malignant tumor phenotype by Hsa_circ_0000566 knockdown was rescued by overexpression of HIF-1α, as indicated by the results of CCK-8, EdU, Transwell, colony formation, and apoptosis assays (Fig. 6H-L). These findings indicated that the effects of Hsa_circ_0000566 on glycolysis in OS cells under hypoxic stress depend on HIF-1α expression.

### HIF-1α transcriptionally activates Hsa_circ_0000566 and forms the HIF-1α-Hsa_circ_0000566-positive feedback pathway

**Figure 7.**
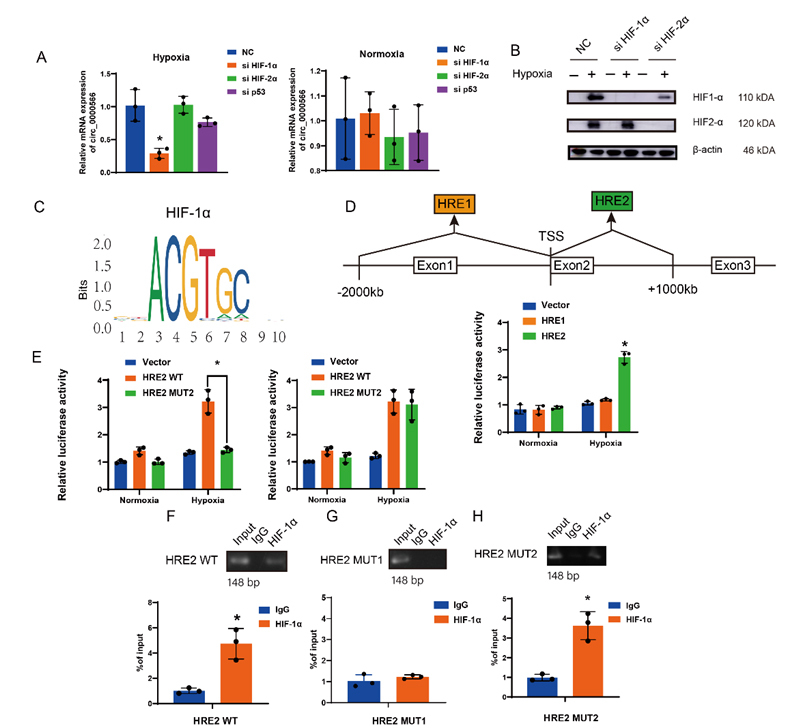
HIF-1α targets and regulates Hsa_circ_0000566 in glycolysis under hypoxic stress. (A-B) Quantitative real-time polymerase chain reaction (qRT-PCR) and western blotting demonstrates that among vital transcription factors such as HIF-1α, HIF-2α, p53, and Hsa_circ_0000566, only Hsa_circ_0000566 is induced by HIF-1α under hypoxia. Results are reported as mean ± standard deviation (SD), *p < 0.05, n = 3. (C) The HIF-1 motif. (D) Luciferase report assays identifying the effective binding regions on Hsa_circ_0000566. Results are reported as mean ± SD, *p < 0.05, n = 3. (E) Luciferase gene experiment shows that mutant 1 is the binding site that regulates Hsa_circ_0000566 transcription. Results are reported as mean ± SD, *p < 0.05, n = 3. (F-H) ChIP assay indicates that HIF-1α is not bound to the genomic region after mutation of HRE2 mutant 1. Results are reported as mean ± SD, *p < 0.05, n = 3. (I) Schematic diagram of the HIF-1α/Hsa_circ_0000566/HIF-1α loop.

The mechanism by which Hsa_circ_0000566 is upregulated under hypoxic conditions was explored. Hsa_circ_0000566 induction by HIF-1α, HIF-2α, and p53 was examined and only HIF-1α expression induced Hsa_circ_0000566 expression under hypoxic stress (Fig. 7A-B). The JASPAR bioinformatics website was used to predict whether the Hsa_circ_0000566 promoter and exons possessed HIF-1α motifs (Figure 7C). Hsa_circ_0000566 contained two possible binding regions. A two-region HRE luciferase reporter gene plasmid was designed, and it was observed that HIF-1α expression activated HRE2 (Fig. 7D). Two HRE2 mutant luciferase reporter gene plasmids were constructed (Supplementary Fig. 5), and the experiment indicated that HIF-1α expression did not activate HRE2 mutant 1 (Fig. 7E). Simultaneously, a chromatin immunoprecipitation (ChIP) assay showed that HIF-1α no longer bound to the genomic region after an effective HRE2 mutation (Fig. 7F-H). These data demonstrate that HIF-1α expression activated Hsa_circ_0000566 at a transcriptional level.

**Figure 8.**
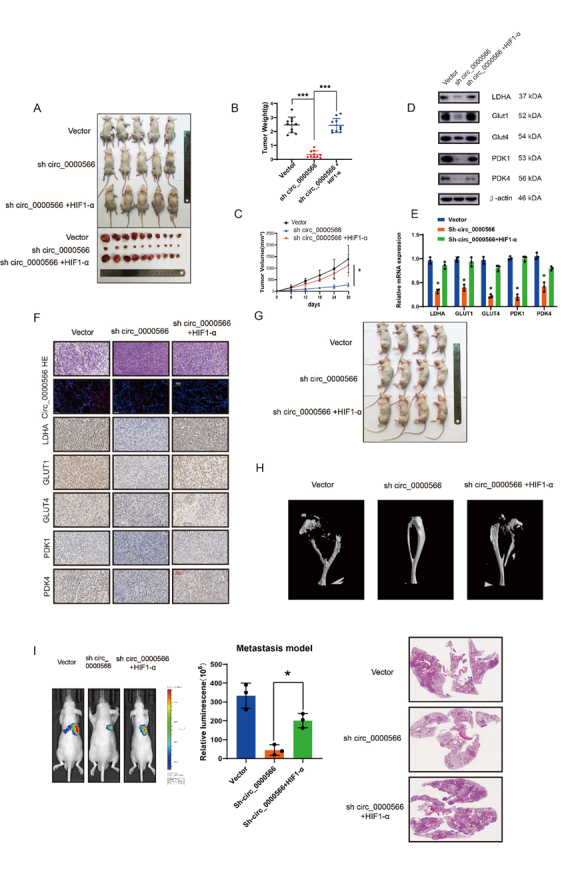
Hsa_circ_0000566 promotes osteosarcoma (OS) glucose metabolism and tumorigenesis progression in vivo. (A) 143B cells stably transfected with Hsa_circ_0000566 knockdown, HIF-1α overexpression, or empty vector plasmids. Nude mice were subcutaneously injected with 1 × 10^7^ cells that were either stable negative controls or those with Hsa_circ_0000566 knockdown, HIF-1α overexpression, or Hsa_circ_0000566 knockdown. Thirty days after injection, the animals were euthanized, and their tumors dissected and photographed. (B) Tumor weight measurements on the same day the mice were euthanized. Results are reported as mean ± standard deviation (SD), *p < 0.05, n = 5. (C) Tumor volumes (ab2/2) were calculated every 6 d from the day after the mice were injected with stable OS cells. (D-E) Western blotting and quantitative real-time polymerase chain reaction (qRT-PCR) exhibit the expression levels of the genes involved in glycolysis metabolism. Results are reported as mean ± SD, *p < 0.05, n = 3. (F) Fluorescence in situ hybridization (FISH), hematoxylin and eosin (H&E) staining, and immunohistochemistry (IHC) analysis indicate the OS organization in mice and relative GLUT1, GLUT4, PDK1, PDK4, and LDHA protein levels in tumors from different groups. (G) In situ tumor formation experiment reveals that HIF-1α overexpression recovered Hsa_circ_0000566 knockdown-induced tumor attenuation. Results are reported as mean ± SD, *p < 0.05, n = 4. (H) Micro-computed tomography (CT) indicates the functions of HIF-1α and Hsa_circ_0000566 knockdown in bone loss. (I) H&E staining of lung metastasis. In mice injected in the tail vein with various stable 143B cells, lung metastasis was detected using an in vivo bioluminescence imaging system. Results are reported as mean ± SD, *p < 0.05, n = 5.

### Hsa_circ_0000566 promotes OS tumorigenesis in vivo

Subcutaneous xenograft models were developed and used to investigate the function of Hsa_circ_0000566 in vivo (Fig. 8A). Transfection was performed using a control or sh-Hsa_circ_0000566. Co-transfection was performed using sh-Hsa_circ_0000566 and HIF-1α. Hsa_circ_ 0000566 downregulation and HIF-1α upregulation were confirmed by qRT-PCR (Supplementary Fig. 6A-B). Hsa_circ_0000566 significantly inhibited OS tumor growth compared to the control; however, OS tumor size was recovered in the group co-transfected with sh-Hsa_circ_0000566 and HIF-1α (Fig. 8B-C). Figure 8D and 8E show that sh-Hsa_circ_0000566 OS tumors had lower glucose transporter (GLUT) and LDHA expression levels compared to those of the control. Additionally, HIF-1α transfection upregulated the expression levels of both GLUT1 and LDHA. Immunohistochemistry (IHC), hematoxylin and eosin (H&E) staining, and FISH were used to evaluate GLUT1 and LDHA expression in OS tumors (Fig. 8F). Thus, the in vitro experiment results were confirmed in vivo. We constructed an orthotopic xenograft tumor model (Figure 8G). Micro-CT showed that Hsa_circ_0000566 knockdown inhibited the growth of 143B cell tumors following tibial platform destruction (Fig. 8H). In contrast, HIF-1α expression aggravated tibial platform destruction. To explore the metastatic potential of Hsa_circ_0000566 in vivo, OS cells were generated with or without stable Hsa_circ_0000566 inhibition or with Hsa_circ_0000566 inhibition and HIF-1α overexpression, after which the cells were labeled with a luminescent dye and injected into nude mice via the tail vein (Fig. 8I). Luminescence revealed lower lung metastasis in the sh-Hsa_circ_0000566 group than in the control group, four weeks post-injection. Hsa_circ_0000566 and HIF-1α co-transfection augmented metastatic potential, as suggested by the in vivo bioluminescence imaging data. The pulmonary metastasis H&E results were similar to those obtained using bioluminescence imaging (Fig. 8I). These data confirmed that Hsa_circ_0000566 may play a critical role in promoting OS tumorigenesis in vivo.

## DISCUSSION

OS is a typical solid tumor that readily undergoes metastasis, is insensitive to radiotherapy and chemotherapy, and presents with a hypoxic microenvironment [40]. Hypoxia significantly accelerates malignant transformation and tumor metastasis, while reducing malignant tumor sensitivity to radiotherapy and chemotherapy [41]. Hence, hypoxia is a major bottleneck in the clinical treatment of malignant solid tumors such as OS. This study revealed that in OS, cyclic RNA-Hsa_circ_0000566 is the main downstream effector of the critical hypoxia transcription factor, HIF-1α. We found that Hsa_circ_0000566 expression was significantly upregulated under hypoxic stress and promoted OS invasion, metastasis, and glycolysis.

CircRNAs have several predominant molecular mechanisms. First, circRNAs contain numerous microRNA (miRNA) binding sites [42]. miRNAs are sponges that indirectly regulate the expression of downstream target genes of miRNAs [43]. Second, circRNAs have at least one protein binding site [44]. Therefore, they also act as protein sponges and can function as protein molecules. Third, certain endogenous circRNAs possess antiviral action, whereas others are associated with immune response elicitation [45, 46]. Exogenous circRNAs stimulate immune signals in mammalian cells by activating the RIG-I pattern recognition receptor [45]. Fourth, although circRNAs are generally non-coding, a few encode polypeptides as part of their regulatory functions [47]. circRNAs are closely associated with the occurrence and development of disease. Therefore, they are considered promising biomarkers and therapeutic targets, along with miRNAs and lncRNAs, and presents a potential focus area for research in clinical disease.

The Warburg effect is one among ten characteristics of tumors [48]. The coding gene protein was the key factor regulating tumor energy metabolism. However, it has been subsequently shown that non-coding RNA is also involved in the reprogramming of tumor energy metabolism [49]. T For instance, the lncRNA, LINC00518, induces radiation resistance in melanomas by regulating HIF-1α expression and modulating glycolysis [50]. In low-oxygen microenvironments, cancer cells obtain their energy primarily by inhibiting aerobic respiration and by promoting glycolysis and the tricarboxylic acid (TCA) cycle [51]. Several transcription factors (TFs) related to glycolysis and metabolism play important roles in tumor anti-apoptosis, drug, and radiation resistance, among other processes [52].

HIF-1α is ubiquitous in mammalian cells and is the only TF that remains active under hypoxic conditions [53, 54]. HIF-1α participates in many cellular signaling pathways and is the transduction center that mediates hypoxic signals [55]. It is expressed during the formation and development of the cardiovascular system, cartilage, and neurula. It regulates cell growth, proliferation, migration, and apoptosis and is involved in a wide range of physiological and pathological processes [56, 57]. HIF-1α and its associated signaling pathways may help clarify the molecular mechanisms of physiological regulation and may help to control disease occurrence and development. Additionally, HIF-1α expression aggravates malignant metastasis in OS cells [58]. The main target of HIF-1α is LDHA, which catalyzes the reduction of pyruvate to lactate [59]. LDHA compensates for the decreased oxidative mitochondrial function and maintains cell survival under hypoxic conditions. In radiotherapy and drug resistance, the HIF-1α/LDHA pathway confers protection against tumors. Our study showed that hypoxia-induced circRNA-Hsa_circ_0000566 mediates glycolysis in OS via HIF-1α regulation. Therefore, circRNAs may be key molecules involved in the regulation of glucose metabolism in OS.

Unlike OS, chondroma is a type of benign bone tumor that tends to occur in tubular bones. In this study, Hsa_circ_0000566 achieved higher expression in OS than in chondroma, according to FISH assays. This finding was additionally validated by qRT-PCR at the mRNA level. Consequently, we inferred that Hsa_circ_0000566 plays a critical role in OS progression.

Our results demonstrate that under hypoxic conditions, HIF-1α is a transcriptional regulator upstream of circRNA-Hsa_circ_0000566, whereas Hsa_circ_ 0000566 binds downstream and stabilizes HIF-1α. These results indicate that HIF-Hsa_circ_0000566 comprises a positive feedback loop that promotes glycolysis, invasion, and metastasis in OS. We also found that Hsa_circ_0000566 inhibits HIF-1α ubiquitination and degradation. Several studies have shown that HIF-1α is a substrate for the VHL complex. Under normoxia, the prolyl hydroxylase domain (PHD) regulates HIF-1α stability, which is in turn recognized by VHL and modified by ubiquitination. We found that Hsa_circ_0000566 binds to both HIF-1α and VHL proteins. However, Hsa_circ_0000566 did not regulate VHL protein expression. Instead, it inhibited the binding of VHL to HIF-1α. Therefore, the Hsa_circ_0000566-VHL combination prevented further binding between HIF-1α and VHL. Our experiment ruled out the possibility of a Hsa_circ_0000566-VHL-HIF ternary complex formation. Using rescue experiments, we demonstrated that the regulation of OS glycolysis, invasion, and metastasis by Hsa_circ_0000566 is mediated by HIF-1α. Hence, Hsa_circ_0000566 is a novel non-coding effector molecule of HIF-1α in glycolysis and may serve as a potential target for OS treatment.

KDM6B-mediated histone LDHA demethylation promotes lung metastasis in OS [60]. As HIF-1α plays an important role in hypoxia and glucose metabolism, cancer treatment strategies that may help inhibit HIF-1α activity will have broad application prospects. Inhibitors of glycolysis-related genes, such as PDK1 and LDHA, located downstream of HIF-1α, could decelerate tumor growth [61]. Li Z () reported that PDK1 mitogenesis was upregulated in OS cells [62]. Thus, PDK1 depression in OS may constitute a critical oncogenic cascade. Jiang et al. () found that LDHA expression was upregulated in OS and promoted tumor invasion and migration [60]. Our subcutaneous xenograft, orthotopic mouse, and tail vein lung metastasis models revealed that Hsa_circ_0000566 mediates OS tumorigenesis and lung metastasis via HIF-1α expression.

Based on the data presented in this study, we propose the existence of a novel HIF-1α-Hsa_circ_0000566 positive-feedback path. Under normoxic conditions, VHL ubiquitinates and rapidly degrades the hydroxylated HIF-1α. In the hypoxic environment of OS, HIF-1α strongly induces Hsa_circ_0000566. Hsa_circ_0000566 then inhibits HIF-1α binding to VHL, hinders HIF-1α protein degradation, upregulates the expression of glycolysis-related enzymes downstream of HIF-1α, and promotes glycolysis by binding to HIF-1α and VHL. Therefore, Hsa_circ_0000566 is a potential therapeutic target for treating OS.

### Conclusions

Our study confirmed that Hsa_circ_0000566 is upregulated in both OS cell lines and tissues. In addition, we discovered that Hsa_circ_0000566 contributes to OS progression and glycolysis by interacting with HIF-1α and VHL under hypoxic conditions.

## Supplementary Materials

The Supplementary data can be found online at: www.aginganddisease.org/EN/10.14336/AD.2022.0826.

## Data Availability

All data pertaining this research are accessible from the authors upon reasonable request.
