# Supplementary material for: Positive Feedback Regulation of Circular RNA Hsa_circ_0000566 and HIF-1α promotes Osteosarcoma Progression and Glycolysis Metabolism
Source: Aging Dis. 2023 Apr 1;14(2):529–47. doi: 10.14336/AD.2022.0826 (PMC10017158; doi:10.14336/AD.2022.0826)
Supplement: Supplementary file 1 — The Supplementary data can be found online at: www.aginganddisease.org/EN/10.14336/AD.2022.0826. [file AD-14-2-529-s.pdf]

SUPPLEMENTARY DATA

Positive Feedback Regulation of Circular RNA Hsa_circ_0000566 and HIF-1α promotes Osteosarcoma Progression and Glycolysis Metabolism

Shuying Shen[1,2 #], Yining Xu[1,2#], Zhe Gong[1,2#], Teng Yao[1,2#], Di Qiao[1,2], Yizhen Huang[1,2], Zhenlei Zhang[1,2], Jun Gao[1,2], Haonan Ni[3], Zhanping Jin[4]， Yingchun Zhu[4]， Hongfei Wu[5], Qingxin Wang[5], Xiangqian Fang[1,2*], Kangmao Huang[1,2*], Jianjun Ma[1,2*]

© 2022. Shen S et al. Published online at http://www.aginganddisease.org/EN/10.14336/AD.2022.0826

SUPPLEMENTARY DATA

Supplementary Table 1. The primers used in the Quantitative real-time PCR.

Name	Sequence (5′→ 3′)
Hsa_circ_0006430	
Forward	ACAGTGACAGGGGATCATCG
Reverse	AGGGAGCGTTTCCAATCCAA
Hsa_circ_0098199	
Forward	CTCTGCTGTGAGGGCAACTC
Reverse	CAAAGCTGATGAGCCAGGGT
Hsa_circ_0000566	
Forward	TAGGAAGTAAGGATGATGGC
Reverse	CACGAGGCATTTTCACTTTG
Hsa_circ_0005211	
Forward	CCCCGAGTCTGGTAAAGCATC
Reverse	GGTCAGAATGGCATACCTCTGT
Hsa_circ_0036649	
Forward	GATTGGACATTTCAGTCTAGAAGGC
Reverse	CCCGTTCCTCACCACTGAAT
Hsa_circ_0002599	
Forward	TTCACTGAAGCCCCTCCG
Reverse	CCGCACTTCCAGTTCTCTCT
Hsa_circ_0047378	
Forward	AGTGTCCCCTGTGTCAAGAATC
Reverse	TTCTACAGAAACACAGGAACTATCT
Hsa_circ_0007509	
Forward	CACGTTCGAAAGGTCTGTGC
Reverse	AAACTCCGGGCCACCATTTG
Hsa_circ_0008821	
Forward	CCTCTCAGATATTAGGACACTGGG
Reverse	CAGCAGTGTCCCAGATGAGG
Hsa_circ_0050334	
Forward	CCGGAAAGAACGTGGTCACT
Reverse	ATCAGGCAAGGTGCTGAGTC
Hsa_circ_0003810	
Forward	CCACCCAGCATTGGTGAAGT
Reverse	TTGTACTCCAGGAACACTTTGAG
Hsa_circ_0005762	
Forward	TGGAGGGTATGGTCAGTTGG
Reverse	TCGAGACCCCAAGCATACAC
Hsa_circ_0000633	
Forward	CGGCACAACTCCTTGGTCTC
Reverse	CCATTATGGAAAGGCCGGGT
Hsa_circ_0007287	
Forward	AGACAGAGCACGAAAGGCAT
Reverse	CATGGCTGGCATTTCCTCAAC
Hsa_circ_0000741	
Forward	TCCTCGCATGATTGTCACCC
Reverse	TTCCACCCGCTCCAGGAAG
Hsa_circ_0050461	
Forward	TATCAACCAGGCCTTTGCCA
Reverse	AAGAGCTTCCAGCTACTTGTCT
Hsa_circ_0004594	
Forward	GGGTACGCTGGCTTCATCAT
Reverse	ACGGTGCACCCGCCG
Hsa_circ_0007935	
Forward	CAGTAATGAACACTGGGCAGC
Reverse	TTGCAAGCTTCATGCAATGGT
Hsa_circ_0005762	
Forward	ATATGTCTGTCAGGATAGAGATTCC
Reverse	TTACCATGCTCTGTCGCTGG
Hsa_circ_0007291	

© 2022. Shen S et al. Published online at http://www.aginganddisease.org/EN/10.14336/AD.2022.0826

Forward	CTCAATGGCGGTATGTGGGT
Reverse	TGTCTCTCAGCACGTGGTTC
Hsa_circ_0127512	
Forward	ACGACCCAGGTTGGTTGAAG
Reverse	AGAGCTGGCCCTATATTATTTTCC
GAPDH	
Forward	TCAAGATCATCAGCAATGCC
Reverse	CGATACCAAAGTTGTCATGGA
Beta Actin	
Forward	CATGTACGTTGCTATCCAGGC
Reverse	CTCCTTAATGTCACGCACGAT
U6	
Forward	CTCGCTTCGCRCAGCACA
Reverse	AACGCTTCACGAATTTGCGT
HIF-1α	
Forward	GAACGTCGAAAAGAAAAGTCTCG
Reverse	CCTTATCAAGATGCGAACTCACA
VHL	
Forward	GCAGGCGTCGAAGAGTACG
Reverse	CGGACTGCGATTGCAGAAGA
LDHA	
Forward	ATGGCAACTCTAAAGGATCAGC
Reverse	CCAACCCCAACAACTGTAATCT
PDK1	
Forward	CTGTGATACGGATCAGAAACCG
Reverse	TCCACCAAACAATAAAGAGTGCT
PDK4	
Forward	GGAGCATTTCTCGCGCTACA
Reverse	ACAGGCAATTCTTGTCGCAAA
GLUT1	
Forward	GGCCAAGAGTGTGCTAAAGAA
Reverse	ACAGCGTTGATGCCAGACAG
GLUT4	
Forward	TGGGCGGCATGATTTCCTC
Reverse	GCCAGGACATTGTTGACCAG

Supplementary Table 2.1. Clinical features of Osteosarcoma applied in experiments.

Features	No. of cases
**Age at diagnosis**	
＜18	8
≥18	4
**Gender**	
Male	6
Female	6
**Clinical classfication**	
Osteosarcoma	8
Osteosarcoma of extraosseous soft tissue	1
Parosteal Osteosaroma	1
**Distant metastasis**	
Absent	3
Present	9
**Tumour size (cm)**	
＜5cm	4
＞5cm	6

© 2022. Shen S et al. Published online at http://www.aginganddisease.org/EN/10.14336/AD.2022.0826

SUPPLEMENTARY DATA

Supplementary Table 2.2. Clinical features of Chondroma applied in experiments.

Features	No. of cases
**Age at diagnosis**	
＜18	5
≥18	7
**Gender**	
Male	7
Female	5
**Clinical classfication**	
enchondroma	8
periosteal chondrolila	4
**Distant metastasis**	
Absent	12
Present	0
**Tumour size (cm)**	
＜5cm	4
＞5cm	6

Statement: our project was authorized by the Ethics Committee of the Sir Run Run Shaw Hospital. The ethic number was 20210218-30.

© 2022. Shen S et al. Published online at http://www.aginganddisease.org/EN/10.14336/AD.2022.0826

SUPPLEMENTARY DATA

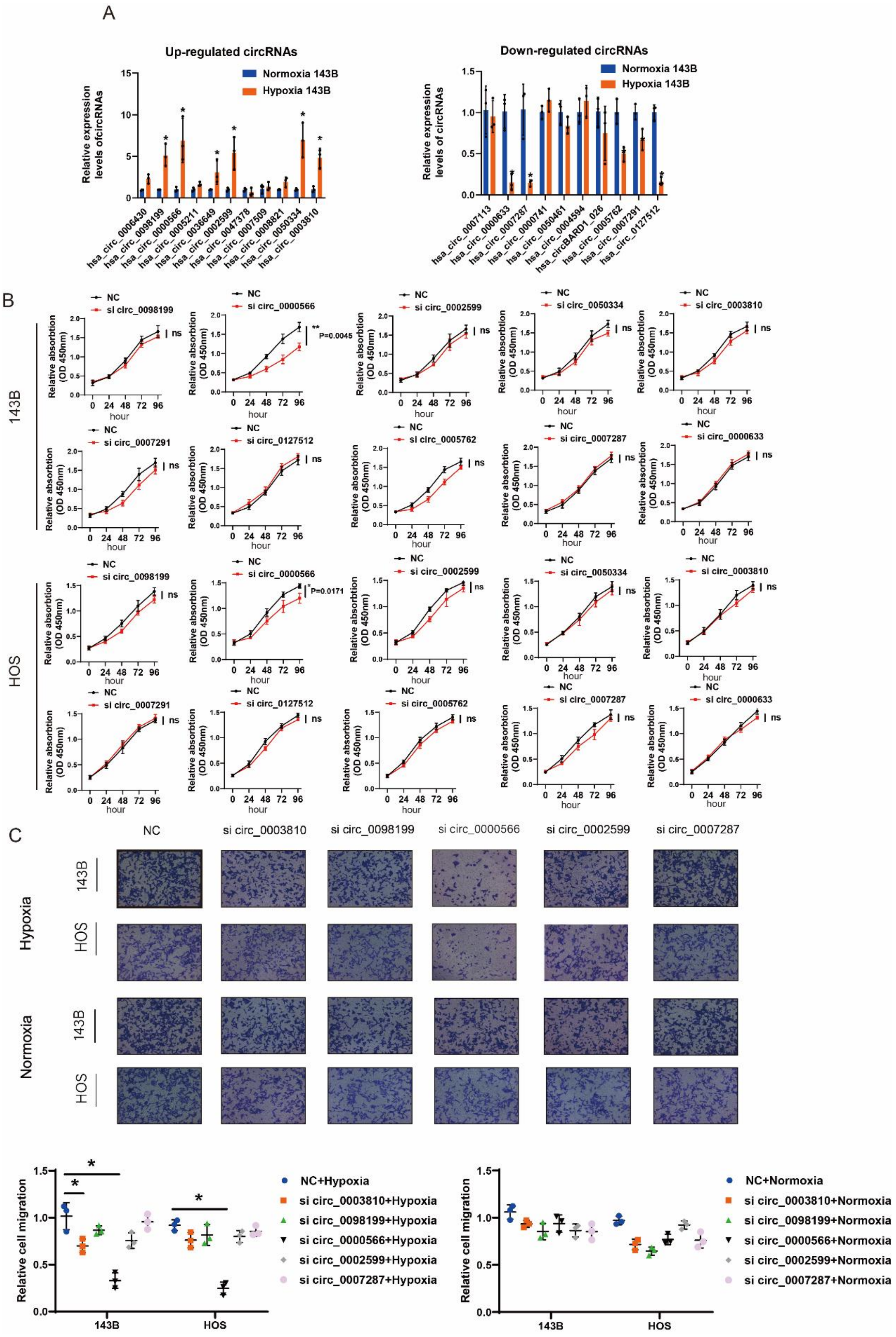

© 2022. Shen S et al. Published online at http://www.aginganddisease.org/EN/10.14336/AD.2022.0826

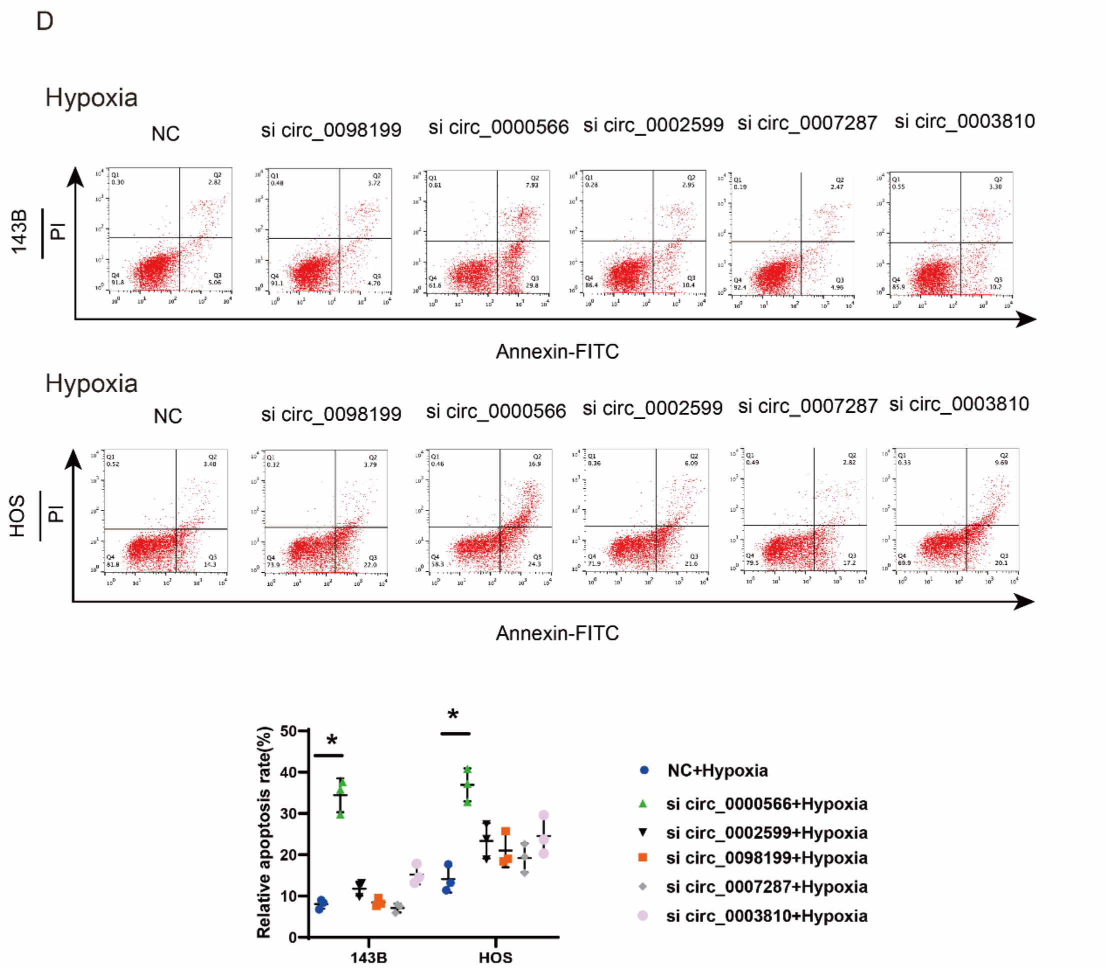

Supplementary Figure S1. The hypoxia-response circRNAs selection in vitro. (A) Five upregulated and five downregulated circRNAs were selected using qRT-PCR. Data were shown as mean ± SD. *p < 0.05. (n=3). **(B)** CCK-8 assays were performed to detect the effect of circRNAs in OS cells. Data were shown as mean ± SD. Ns represents no sense. *p < 0.05. (n=3). **(C)** Transwell migration experiment was employed to examine the impact of candidate circRNAs on OS cells. Data were shown as mean ± SD. *p < 0.05. (n=3). **(D)** Flow cytometry analysis was employed to pick out functional and hypoxia-response circRNAs from candidate circRNAs. Data were shown as mean ± SD. *p < 0.05. (n=3).

© 2022. Shen S et al. Published online at http://www.aginganddisease.org/EN/10.14336/AD.2022.0826

SUPPLEMENTARY DATA

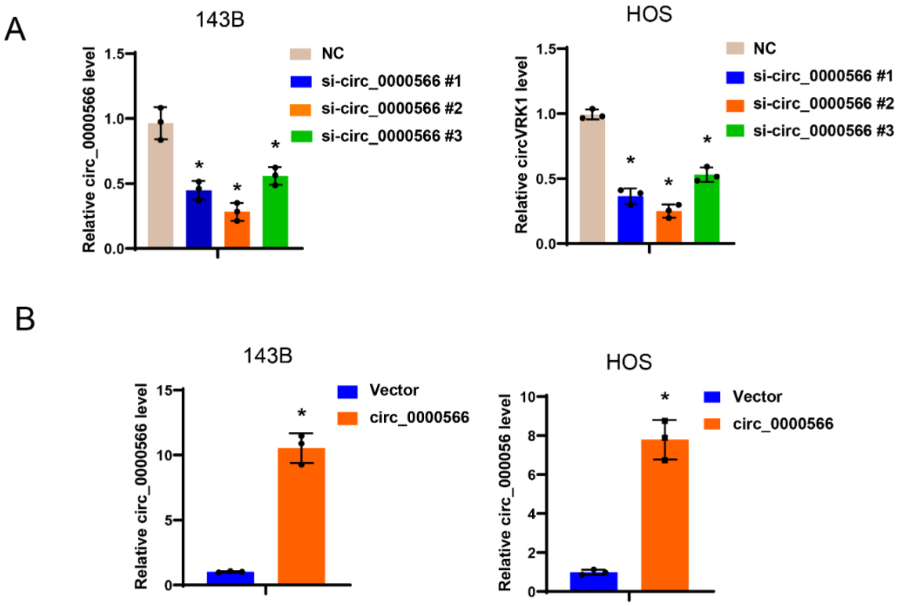

Supplementary Figure S2. The knockdown efficiency of hsa_circ_0000566. (A) The expression levels of hsa_circ_0000566 in 143B and HOS cells treated by transfection of hsa_circ_0000566 silencing or control siRNAs were assessed by qRT-PCR. Data were shown as mean ± SD. *p < 0.05. (n=3). **(B)** The expression levels of hsa_circ_0000566 in 143B and HOS cells treated by transfection of hsa_circ_0000566 overexpression or vector. were assessed by qRT-PCR. Data were shown as mean ± SD. *p < 0.05. (n=3).

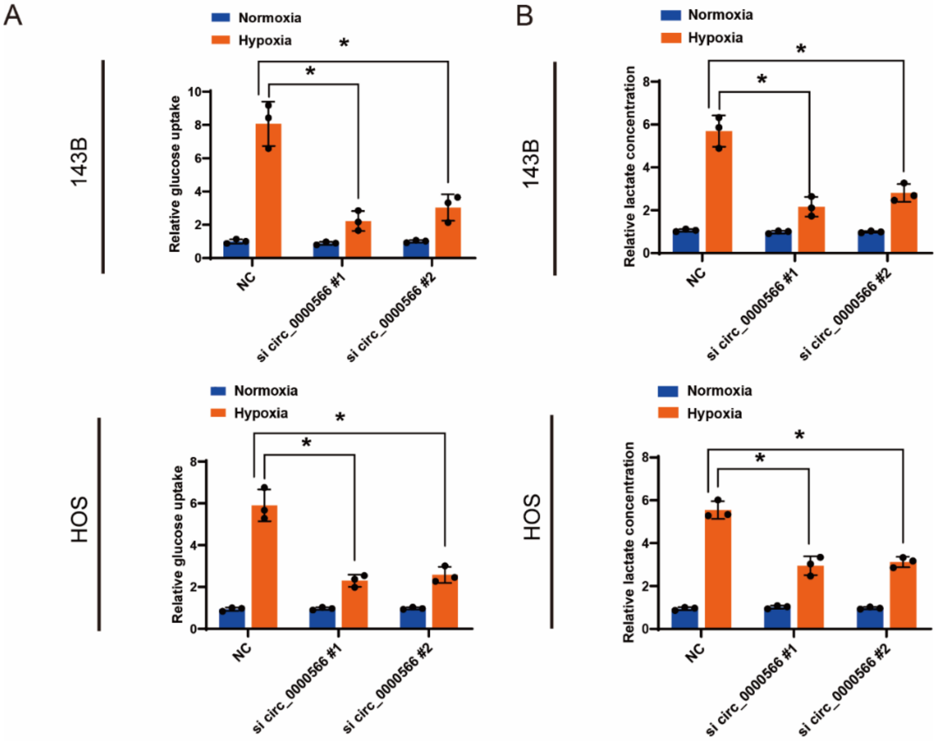

Supplementary Figure 3. Hsa_circ_0000566 promotes glycolysis in osteosarcoma. (A) The glucose uptake in 143B and HOS cells treated by transfection of hsa_circ_0000566 silencing or control siRNAs were assessed. Data were shown as mean ± SD. *p < 0.05. (n=3). **(B)** The lactate production of 143B and HOS cells treated by transfection of hsa_circ_0000566 overexpression or silencing or control siRNAs were assessed. Data were shown as mean ± SD. *p < 0.05. (n=3).

© 2022. Shen S et al. Published online at http://www.aginganddisease.org/EN/10.14336/AD.2022.0826

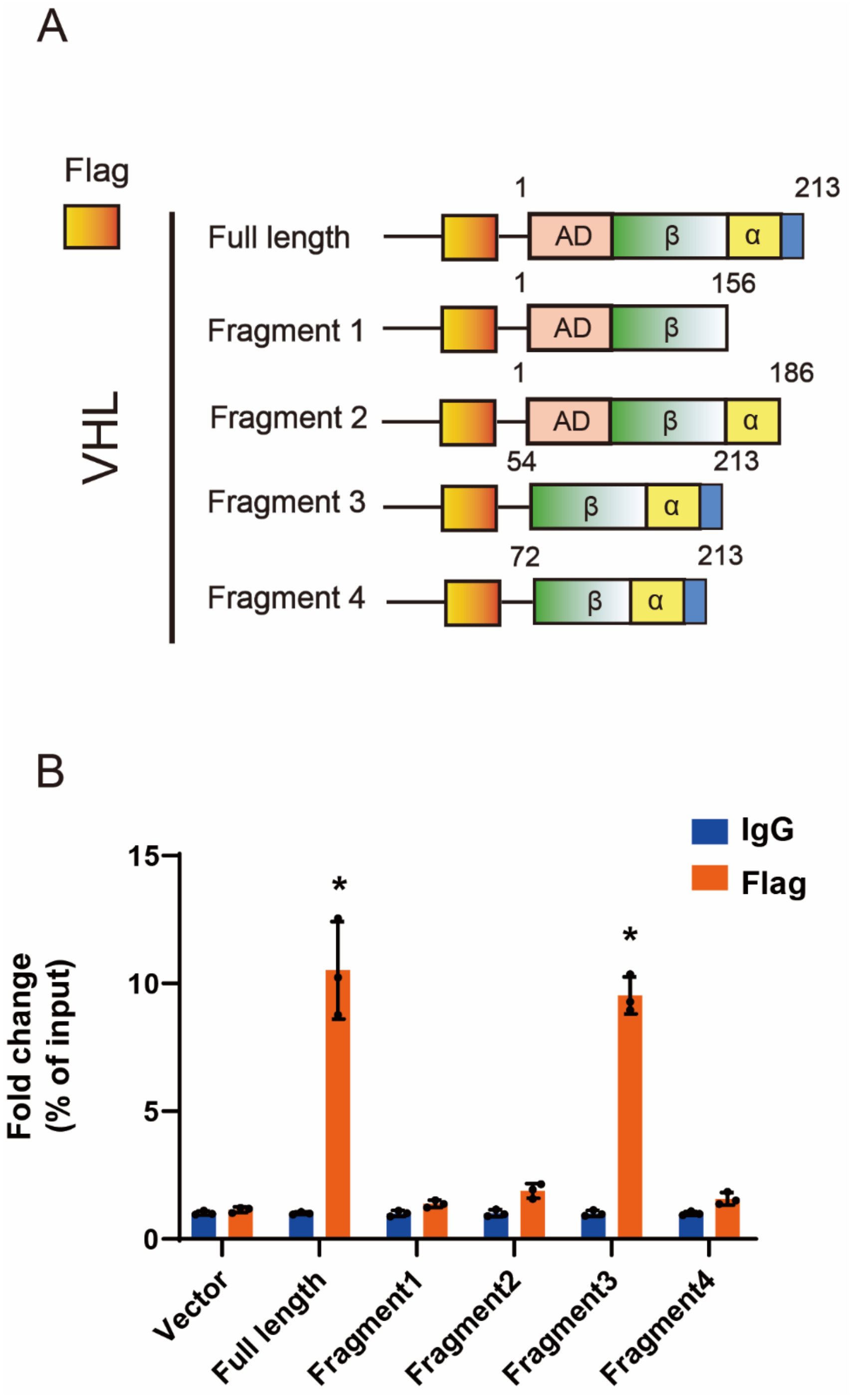

Supplementary Figure 4. Deletion mutant experiment of VHL. (A) A schematic of full length and deletion mutants of VHL. (B) RIP experiment was used to verify specific VHL regions interacting with hsa_circ_0000566. Results are reported as mean ± SD, *p < 0.05, n = 3.

© 2022. Shen S et al. Published online at http://www.aginganddisease.org/EN/10.14336/AD.2022.0826

SUPPLEMENTARY DATA

A

HRE1

ACACCTGAGGGCACTTGAAGCCCAGTGTGAAGTGACTTGATCAAGATTAC
CCAACCAGCTGGTGTCATACCTGCAATCCAGGTCTTTTGATTCCAGTCAG
TACTCTTTTCACAATAAAACACTGCTTTCTCTGAATGTTATGTTAAACTT
ATGTGTCAGTGAGAAAAATGAATCAAGATTCATCTCCTAAAGGATAGCTA
ATGGGAAAGAAAGCTTAGGAATTCTGGGTGTCACCAGCCTTGCCCAGATG
ATTAAGCCACAGTTTTTCCTGTCACTCTTGAAAGTTGTGCTCCTTCCTCA
CAGTGACTTCCATAATTGTGGAGCATGTTGGTGAGGTCTTGGAGCCAGGT
GGTCACCGGTGCAATTTTCTGTTGTTCCACCACTTACCAGCTTGCTGAGC
CTTAGTTTCCTCGAAATGGAGGTGATAATGTAATCTTCACAGAGTCATGA
GGATGGATGGGAACCTGTGTAAAGCATTTACTAGCATGTCCCGGGAACCT
TTGCCCCTTCCCCTTGTGCTCAGTTGCCTTATGTTTTCAATCTCTGCACT
GTGTTCTATCATATCGGTATTGACCTTAATAAACTCTTCATTTTGTTCCT
ATAAAATGTGCATTCTAATTGGATGCACCTTTCCCTC

HRE2

GTGAAAATGCCTCGTGTAAAAGCAGCTCAAGCTGGAAGACAGAGCTCTGC
AAAGAGACATCTTGCAGAACAATTTGCAGTTGGAGAGATAATAACTGACA
TGGCAAAAAAGGAATGGAAAGTAGGATTACCCATTGGCCAAGGAGGCTTT
GGCTGTATATATCTTGGTAAGTGTGTGACTGCTTCTAATGATCAATCCAA
AGATTTATATGTTTTCTTATGAAAATGGTTTCTCATTTATGAGCTGTTAT
GGGATGTTCCTAATAATCTGCAGTCAACTTAATAGTTTCTGATTAAGCAA
AAATGCATCTCTGACGTAGGAGTGGAAATTGTTAATGCAGTCACAACAGA
AAAGGAGGATAAAATAGCTGTAATACTATCATTAGTGAGCACTAGGACTT
TAGATTTTTAAGATACTTTATATAATCTTAATCTCTTTTGAGACCCAAAG
AAACCCATTTACGTATCTTTCCCCTCCCACCTCCACCTATGTGGGATTTA
TTCCCTCCGTGTT

B

HRE2 MUT1

(CGTG → AAAA)

HRE2 WT
...ATGCCTCGTGTAAAAG...

HRE2 MUT1
...ATGCCTAAAATAAAAG...

HRE2 MUT2

(CGTA → AAAA)

HRE2 WT
...CTCTGACGTAGGAGTGG...

HRE2 MUT2
...CTCTGAAAAAGGAGTGG...

Supplementary Figure 5. The combination of HIF-1α and hsa_circ_0000566. Prediction of the binding position of HIF-1α to the hsa_circ_0000566 (JASPAR). Hsa_circ_0000566 sequence labeling HIF-1α-binding site (blue) and the mutated nucleotides (red).

© 2022. Shen S et al. Published online at http://www.aginganddisease.org/EN/10.14336/AD.2022.0826

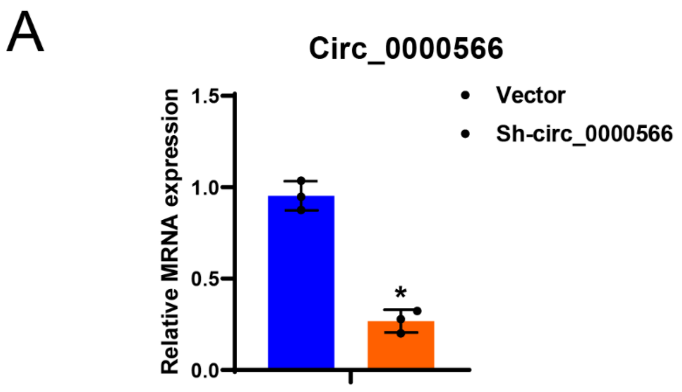

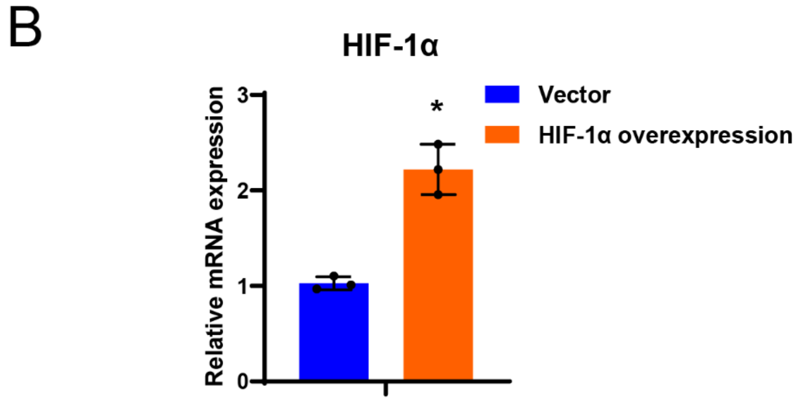

Supplementary Figure 6. The knockdown efficiency of sh-hsa_circ_0000566 and the overexpression efficiency of HIF-1α. A. The expression levels of hsa_circ_0000566 in osteosarcoma cells transfected with sh-hsa_circ_0000566 were detected by qRT-PCR. Data were shown as mean ± SD. *p < 0.05. (n=3). B. The expression levels of HIF-1α in osteosarcoma cells transfected with HIF-1α overexpression were detected by qRT-PCR. Data were shown as mean ± SD. *p < 0.05. (n=3).

© 2022. Shen S et al. Published online at http://www.aginganddisease.org/EN/10.14336/AD.2022.0826
